# The metabolic characteristics and environmental adaptations of the intertidal bacterium *Palleronia* sp. LCG004

**DOI:** 10.3389/fmicb.2024.1469112

**Published:** 2024-11-22

**Authors:** Zekai Wang, Jiahua Wang, Xi Yu, Hongcai Zhang, Jie Liu, Junwei Cao, Jiasong Fang, Zengfu Song, Li Zhang

**Affiliations:** ^1^Shanghai Engineering Research Center of Hadal Science and Technology, College of Marine Sciences, Shanghai Ocean University, Shanghai, China; ^2^Laboratory for Marine Biology and Biotechnology, Qingdao National Laboratory for Marine Science and Technology, Qingdao, China; ^3^College of Fisheries and Life Sciences, Shanghai Ocean University, Shanghai, China; ^4^National Demonstration Center for Experimental Fisheries Science Education, Shanghai Ocean University, Shanghai, China; ^5^China-ASEAN Belt and Road Joint Laboratory on Mariculture Technology (Shanghai), Shanghai Ocean University, Shanghai, China

**Keywords:** intertidal zone, *Palleronia*, environmental adaptations, metabolic versatility, genomic comparison

## Abstract

The intertidal zone, a dynamic interface of marine, atmospheric, and terrestrial ecosystems, exposes microorganisms to rapid shifts in temperature, salinity, and oxidative stress. Strain LCG004, representing a novel *Palleronia* species, was isolated from the Lu Chao Harbor’s intertidal seawater in the Western Pacific Ocean. The genome of the organism reveals its metabolic versatility, enabling the utilization of various organic substrates—ranging from organic acids, amino acids, to sugars, and encompassing complex carbohydrates—as well as adept handling of inorganic nutrients, thereby highlighting its significant role in the cycling of nutrients. The strain is equipped with multiple osmoprotectant transporters, deoxyribodipyrimidine photo-lyase, and a comprehensive antioxidant defense system, featuring with multiple catalases, peroxidases, and superoxide dismutases, enabling it to withstand ever-changing environmental conditions, UV radiation, and oxidative challenges. Notably, LCG004 exhibited enhanced growth and cell aggregation under oligotrophic conditions, promoted by light exposure, underscoring the significant influence of light on its morphological and physiological attributes. This study elucidates strain LCG004’s metabolic characteristics and ecological potential, and offers insights into its contributions to biogeochemical cycles and survival strategies in one of nature’s most fluctuating environments.

## 1 Introduction

The intertidal zone is a highly dynamic and challenging environment where microorganisms are subjected to a multitude of stressors (Kafanov et al., 2004; Wang et al., 2017). Situated at the intersection of marine, atmospheric, and terrestrial ecosystems, this region is characterized by extreme fluctuations in conditions such as temperature, salinity, moisture and dissolved oxygen (Stillman, 2001; Kuklinski and Barnes, 2008), which are exacerbated by the daily ebb and flow of tides. Microorganisms there must contend with desiccation, intense ultraviolet radiation (UV) during low tide, and potentially damaging reactive oxygen species (ROS) generated under both anoxic and oxidative conditions (Bertness et al., 2004; Skarphéoinsdóttir et al., 2005; Tikhonenkov et al., 2015). The intertidal environment also imposes physical challenges, such as the mechanical stress from wave action and the need to adhere to surfaces amidst the shifting substrates (Lemos et al., 2018). To survive these harsh conditions, microorganisms have evolved a suite of adaptive mechanisms, including the production of osmoprotectants to combat osmotic stress, the synthesis of heat shock proteins to manage temperature extremes, and the deployment of antioxidant enzymes to mitigate oxidative damage (Glenn et al., 1999; Stillman, 2001; Song et al., 2008). These adaptations are critical for the resilience and survival of microbial communities in the face of the intertidal zone’s ever-changing environmental conditions (Somero, 2010; Foo and Byrne, 2016). However, the full extent of these adaptations and the specific mechanisms by which microorganisms cope with the intertidal environments remain areas of ongoing investigations.

The genus *Palleronia* belonging to the *Alphaproteobacteria* was first characterized by Martínez-Checa et al. (2005), with the description of a novel, moderately halophilic bacterium, designated as *P. marisminoris* B33*^T^* (= CECT 7066*^T^* = LMG 22959*^T^*). B33*^T^* was isolated from a hypersaline soil bordering a saline saltern on the Mediterranean seaboard in Murcia (Spain) and demonstrated the ability to produce exopolysaccharides (Martínez-Checa et al., 2005). Subsequently, many *Palleronia* species of the genus have been successively identified, e.g., *P. abyssalis* CECT 8504*^T^* from the Matapan Vavilov Deep canyon at a depth of 4,908 meters (Albuquerque et al., 2015), *P. rufa* MOLA 401*^T^* from marine waters in the southwest lagoon of New Caledonia (Barnier et al., 2022); *P. sediminis* SS33*^T^* from offshore areas near Weihai (Sun et al., 2021). Additionally, several species previously classified under the genera *Hwanghaeicola*, *Maribius*, and others were reassigned to the genus *Palleronia* based on their genetic relatedness and phenotypic characteristics (Kim et al., 2010). Although many strains of this genus have been isolated, there has been no systematic research on their genomic characteristics, metabolic potential, ecological functions, or mechanisms of environmental adaptability.

This study introduces a novel bacterial strain, LCG004, derived from the East China Sea intertidal zone. It is taxonomically identified as a member of the *Palleronia* genus, and its genomic sequence is presented as the second completed genome within this genus. Through sophisticated genomic analyses, metabolic network reconstruction, and biological assays, we have delineated the metabolic profiles and ecological potentials of LCG004. Additionally, given that LCG004 originates from ever-changing intertidal environment, whereas many strains of this genus come from environments with less fluctuation (e.g., *P. marisminoris* B33*^T^* from saltern, *P. salina* DSM 26892*^T^* from seawater in lagoon of New Caledonia and *P. abyssalis* CECT 8504*^T^* from deep marine water at 4,908 m), we employed genomic comparison to investigate LCG004’s adaptability to intertidal environments (Albuquerque et al., 2015). This work not only broadens our perspective on the metabolic diversity within the *Palleronia* genus but also sheds light on the intricate survival strategies that microbes harness to navigate the tumultuous intertidal ecosystems.

## 2 Materials and methods

### 2.1 Sample description and bacterial isolation

Surface seawater samples were collected in the intertidal zone of Lu Chao Harbor of the East China Sea (30.82°N, 121.92°E) in December 2022. The samples were filtered with a pore size of 0.2 μm. The microbes on filter membranes were eluted, centrifuged (5,000 rpm/min) and resuspended using artificial seawater (ASW) for three times. Then, the resuspension was cultured on MB 2216E-agar plates (1,000 mL seawater, 5 g Peptone, 1 g Yeast Extract, 15 g Agar, pH 7.6–7.7) at 28°C for two weeks. Finally, the colonies were picked and purified via streaking inoculation. The strain LCG004 (= MCCC 1K08952) was routinely cultivated on 2216E medium under aerobic conditions and stored at −80°C in liquid medium supplemented with 20% (v/v) glycerol.

The artificial seawater (ASW) used in this study contained 52 g/L NaCl, 15 g/L Agar, 10 g/L MgCl_2_⋅6H_2_O, 8 g/L Na_2_SO_4_, 2.8 g/L CaCl_2_⋅2H_2_O, 1 g/L KCl, 0.6 g/L NH_4_Cl, 0.2 g/L KH_2_PO_4_, 2 mM NaHCO_3_, which was autoclaved and supplemented with mixed vitamins and trace elements (1:1000) sterilized using 0.2 μm filter. The trace elements contained 30 mg/L of FeCl_3_⋅6H_2_O, 2 mg/L of MnCl_2_⋅4H_2_O, 0.23 mg/L of ZnSO_4_⋅7H_2_O, 0.2 mg/L of CoCl_2_⋅6H_2_O, 0.1 mg/L of Na_2_MoO_4_⋅2H_2_O, 0.2 mg/L of Na_2_SeO_3_ and 0.2 mg/L of NiCl_2_⋅6H_2_O, while the supple-mentary vitamin mixture included 1.8 g/L of thiamine, 1 g/L of myoinositol, 100 mg/L of pyridoxine, 98.4 mg/L of nicotinic acid, 80 mg/L of 4-aminobenzoic acid, 30 mg/L of pantothenic acid, 1 mg/L of folate, 1 mg/L of cobalamin and 0.1 mg/L of biotin. Both trace elements and vitamin mixture were filtrated through 0.1 μm filters before their addition.

To determine the growth curves of strain LCG004, we initially cultivated it in MB 2216E medium for approximately 24 h at 28°C to ensure the culture was in the logarithmic phase. Subsequently, we introduced a small volume of the culture broth into the medium designated for growth curve analysis, and adjusted the initial optical density (OD) to approximately 0.05. The growth curves at different temperatures were measured using MB 2216E medium (pH = 7.2), with temperatures of 4, 10, 15, 20, 25, 28, 32, 37, and 40°C. The pH growth curves were measured by adjusting the pH of MB 2216E medium to 4, 5, 6, 7, 8, 9, and 10 using HCl or NaOH, with the cultivation temperature set at 28°C. To determine the range of NaCl required for the growth of strain LCB004, we first prepared a solution of MB 2216E medium without NaCl. Then, we added a specific amount of NaCl to achieve final concentrations of 0, 2, 4, 6, 8, 10, 12, 16, and 20%. The cultivation temperature was set at 28°C, and the pH was adjusted to 7.2. Each of the above experiments was conducted with three biological replicates, and the measurements of OD_600_ represent the average of three readings for each sample.

### 2.2 Microbial utilization of different substrates and chemotaxonomy

The capacity of the microbe to metabolize different substrates was evaluated in triplicate using 100 mL of ASW liquid medium supplemented with D-xylose, maltose, stachyose, pectin, sodium acetate, peptone or linseed oil, serving as the sole carbon source. The final concentration of each substrate was 5 mM, except linseed oil (1% v/v). Exponential-phase cultures of strain LCG004 were washed three times via centrifugation with ASW liquid medium and then aliquoted into distinct ASW liquid media containing the various substrates for cultivation at 28°C. Aliquots of 50 μL from the bacterial cultures at both 0- and 7-day post-inoculation were, respectively, coated onto marine 2216E agar plates in triplicate and incubated at 28°C. The utilization of different substrates by strain LCG004 was verified by comparing the colony numbers of strain LCG004 on the plates between initial (day 0) and final (day 7) time points. Tests for other physiological or biochemical characteristics were performed using API 20 NE, API ZYM strips (bioMérieux) and GEN III MicroPlates (Biolog) according to the manufacturers’ instructions.

In the context of cellular fatty acid profiling, strain LCG004 was cultivated in MB 2216E medium for a duration of 48 h at a temperature of 28°C. The process of saponification, methylation, and extraction of fatty acids was conducted in accordance with the MIDI (Sherlock Microbial Identification System, version 6.0) standard operating procedure. The analysis of the fatty acid methyl esters was performed using gas chromatography with an Agilent Technologies 6850 instrument and the identification of these compounds was facilitated by the RTSBA6.0 database, which is part of the Microbial Identification System (Athalye et al., 1985).

For the examination of polar lipids in strain LCG004, extraction and separation were carried out on silica gel 60 F254 aluminum-backed thin-layer chromatography plates, which were 10 cm × 10 cm in size and manufactured by Merk with the product number 5,554. The subsequent analysis was conducted following the guidelines established by Minnikin et al. (1984). The chromatographic plates were developed using a two-dimensional solvent system; the first dimension consisted of a mixture of chloroform, methanol, and water in a ratio of 65:24:4 by volume, while the second dimension was a mixture of chloroform, glacial acetic acid, methanol, and water in a ratio of 80:15:12:4 by volume. After the development, the plates were sprayed with a 5% solution of phosphomolybdic acid (w/v) in alcohol and subjected to heating at 160°C for a period of 10 to 15 min to visualize the total lipid content.

The extraction and analysis of respiratory quinones were performed using the methodology outlined by Minnikin et al. (1984) and the high-performance liquid chromatography (HPLC) procedure described by Tindall (1990).

### 2.3 The effects of visible light on the growth dynamics of strain

To elucidate the effect of visible light on the growth dynamics of strain LCG004, we performed triplicate culture experiments using 500 mL of MB 2216E medium and oligotrophic medium (the latter was supplemented with only 1% of MB 2216E organic components). Exponential-phase cultures of strain LCG004 were aliquoted into MB 2216E liquid medium and oligotrophic medium and incubated at 28°C. The cultures were subjected to a photoperiod of 12 h per day and incubated under controlled conditions with and without light. After 72 h of incubation, the difference in biomass between light and dark incubation conditions was determined by measuring the OD_600_ values of the cultures. The organisms were also stained using SYTO 9/PI live/dead bacteria dual strain kit and observed by fluorescence microscope. The effect of visible light on the growth dynamics of strain LCG004 was verified by comparing the bacterial status of strain LCG004 in the control and experimental groups.

### 2.4 Genomic DNA extraction, sequencing, and assembly

MB 2216E medium to was used to cultivate the strain LCG004 to the mid-logarithmic phase (approximately 12 h of cultivation). Then, we used the bacterial DNA extraction kit to extract DNA (Tiangen, China). The genome of strain LCG004 was sequenced using a combination of PacBio RS II Single Molecule Real Time (SMRT) and Illumina HiSeq 2500 platforms by MajorBio (Shanghai Co., Ltd., China).

For Illumina sequencing. approximately 1 μg genomic DNA was sheared into 400–500 bp fragments using a Covaris M220 Focused Acoustic Shearer following the manufacturer’s protocol. Illumina sequencing libraries were prepared from the sheared fragments using the NEXTFLEX™ Rapid DNA-Seq Kit.

For Pacific Biosciences sequencing, an aliquot of 15 μg DNA was spun in a Covaris g-TUBE (Covaris, MA, USA) at 6,000 RPM for 60 s using an Eppendorf 5424 centrifuge (Eppendorf, NY, USS). DNA fragments were then purified, end-repaired, and ligated with SMRTbell sequencing adapters following the manufacturer’s recommendations (Pacific Biosciences, CA, USA). The resulting sequencing library was purified three times using 0.45 × volumes of Agencourt AMPure XP beads (Beckman Coulter Genomics, MA, USA) following the manufacturer’s recommendations. Next, a ∼ 10 kb insert library was prepared and sequenced on one SMRT cell using standard methods.

The data generated from PacBio and Illumina platforms were used for bioinformatics analysis. The complete genome sequence was assembled using both the PacBio reads and the Illumina reads. The original image data are transferred into sequence data via base calling, which is defined as raw data or raw reads and saved as a FASTQ file. A statistic of quality information was applied for quality trimming, by which the low-quality data can be removed to form clean data. The reads were then assembled into a contig using the hierarchical genome assembly process (HGAP) and canu (Koren et al., 2017). The last circular step was checked and finished manually, generating a complete genome with a single seamless chromosome. Finally, error correction of the PacBio assembly results was performed using the Illumina reads using Pilon (Walker et al., 2014). The whole genome sequence of the *Palleronia* sp. LCG004 has been registered on GenBank as chromosomes and plasmids, respectively. The accession numbers are CP136759.1, CP136760.1, CP136761.1, CP136762.1, CP136763.1.

### 2.5 Gene annotation and genomic comparison

The NCBI prokaryotic genome annotation pipeline (Tatusova et al., 2016) was used in ORF prediction and gene annotation. The predicted protein sequences were also aligned with the Clusters of Orthologous Groups of proteins (COG) (Galperin et al., 2021) and TransporterDB 2.0 (Elbourne et al., 2017) databases using the BLASTp software with the following parameters: identity, 50%; query-cover, 80%; and *e*-value 1e-5. The annotation of Kyoto Encyclopedia of Genes and Genomes (KEGG) was assigned with BlastKOALA (Kanehisa et al., 2016). The genomic islands were predicted using IslandViewer 4 (Bertelli et al., 2017).

Protein families of strain LCG004 and phylogenetically related strains were clustered using a local OrthoMCL 2.0.9 (Li et al., 2003) with the following cutoff values: identity, 50%; query coverage, 50%; E value, 1e-10; score, 40; and MCL inflation, 1.5. The protein families employed by only one strain were considered as strain-specific. Average nucleotide identity (ANI) and average amino acid identity (AAI) were calculated using fastANI (Jain et al., 2018) and CompareM,^1^ respectively, with default parameters. The genome-to-genome distance (DDH) was calculated with GGDC 3.0 (Meier-Kolthoff et al., 2022).

### 2.6 Phylogenetic analysis

The 120 conserved bacterial marker genes of the GTDB taxonomy were used to study the phylogeny of strain LCG004 and its related strains. The sequences of 120 marker proteins in the genomes were predicted using GTDB-Tk (database version: Release 07-RS207) (Chaumeil et al., 2019), and separately aligned using Clustal Omega (Sievers and Higgins, 2021). The aligned sequences were manually degapped. If one marker protein was not identified in some genome, we would then add the corresponding number of “-” depending on the length of the sequence after degap. Then, the degapped alignments of each marker protein were tandemly connected (Tang et al., 2021). The phylogenetic tree was constructed using FastTree2 with the neighbor-joining method (Price et al., 2010), and a bootstrap analysis with 1,000 replicates was performed to assess the robustness of the tree. Finally, the phylogenetic tree was plotted using iTOL (Letunic and Bork, 2021).

## 3 Results and discussion

### 3.1 The description of strain LCG004

**FIGURE 1:**
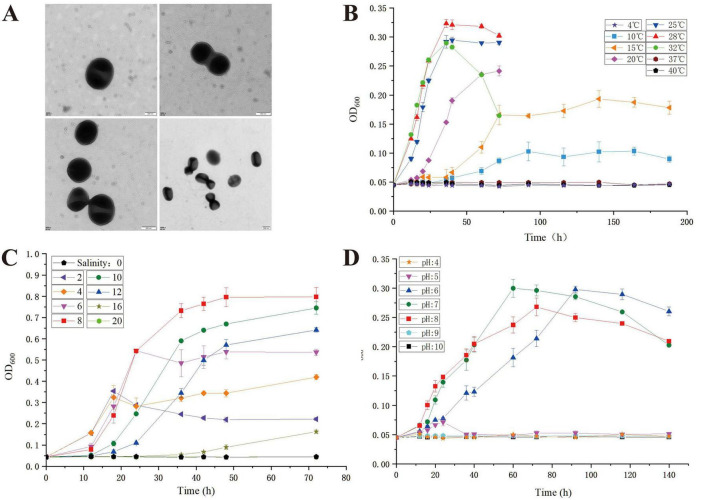
The transmission electron micrograph of strain LCG004 **(A)** and the growth curve of strain LCG004 at different temperatures **(B)**, at different NaCl concentrations **(C)** and at different pH **(D)**.

Strain LCG004 was isolated from the intertidal seawater of the Lu Chao Harbor in the East China Sea (121.9°E, 30.8°N) using agar plates containing marine broth 2216E. The MIGS morphological information of *Palleronia* sp. LCG004 is summarized in Supplementary Table 1. Strain LCG004 is a strictly aerobic, non-spore-forming Gram-negative bacterium. The temperature range for growth is 10–32°C (optimum, 28°C), and its growth occurs at pH 6.0–8.0 (optimum, pH 7.0) and with 2–16 % (w/v) NaCl (optimum, 8 %, Figure 1). Cultivated on marine 2216E agar plates, this strain formed a pink, circular colony with regular and slightly raised edges. Transmission electron microscopy images showed that the cell length of strain LCG004 is 0.68 μm and the width is 0.6 μm. Similar with *P. marisminoris* B33^T^ (Martínez-Checa et al., 2005), *P. soli* CAU 1105^T^ (Kim et al., 2015), *P. abyssalis* 221-F1^T^ (Albuquerque et al., 2015), *P. rufa* MOLA 401^T^ (Barnier et al., 2022), and *P. sediminis* SS33^T^ (Sun et al., 2021), strain LCG004 also lacks flagella (Figure 1).

In physiological and biochemical tests of strain LCG004, nitrate was not reduced to nitrite. Acid production was found with D-glucose was observed, but negative for indole. Alkaline phosphatase, esterase (C4), esterase lipase (C8), leucine arylamidase, acid phosphatase, α-galactosidase, β-galactosidase, α-glucosidase, β-glucosidase, α-mannosidase were present, but lipase (C14), cystine arylamidase, trypsin, α-chymotrypsin, β-glucuronidase, N-acetyl-β-glucosaminidase, α-fucosidase were absent. In carbon source oxidation tests, positive results were obtained for dextrin, D-maltose, D-trehalose, D-cellobiose, sucrose, D-melibiose, α-D-glucose, D-mannose, D-fructose, D-galactose, D-sobitol, D-mannitol, glycerol, pectin, D-galacturonic acid, L-galactonic acid lactone, D-gluconic acid, L-lactic acid, L-malic acid, β-hydroxy-D,L-butyric acid, acetoacetic acid, propionic acid, acetic acid (Supplementary Table 2). Carotenoid was found present in strain LCG004. The phenotypic differences between strain LCG004 and other related strains are shown in the Table 1.

**TABLE 1:** Differential phenotypic between strain LCG004 and four related type strains.

Characteristic	1	2	3	4	5
**Ranges (optimum) for growth:**					
Temperature (°C)	10–32 (28)	4–40 (33)	20–37	15–37 (30)	20–40 (37)
NaCl (w/v, %)	2–16 (8.0)	0.5–15.0 (5.0)	0.0–10.0 (3.0)	0–13 (2–5)	1–11 (3)
pH	6.0–8.0 (7)	6.0–9.5 (7.5–8.0)	5.0–10.0	6.0–8.0 (6.5–7)	4.5–11 (7)
**Enzymic activities:**					
Alkaline phosphatase	+	–	+	−	−
β-Glucosidase	+	W	+	−	−
API 20 NE test:					
Urease	−	−	−	ND	ND
**Oxidation of:**					
Pectin	+	–	ND	ND	ND
Lactose	+	−	−	+	−
L-Histidine	w	+	−	−	ND
Myo-Inositol	w	+	−	−	+
**Acid production from:**					
D-Arabinose	−	−	−	+	−
D-Tagatose	w	+	−	−	−
D-Fucose	w	W	+	+	−
Polar lipids	PG, DPG, 4PL, AL, 3GL, 3L	PG, PC, PL, 2AL, GL, L	PG, PC, GL, AL, GL, 2L	PG, PC, GL, AL, PL, DPG, L	ND
Major fatty acids	C18:1 ω7c, C19:0 cyclo ω8c	C18:1 ω7c, C16:0	C18:1 ω7c, C19:0 cyclo ω8c	C18:1 ω7c, C19:0 cyclo ω8c	C18:1 ω7c

Strains: 1, LCG004; 2, *Palleronia sediminis* SS33^T^ (Sun et al., 2021); 3, Palleronia marisminoris DSM 26347^T^ (Martínez-Checa et al., 2005); 4, Palleronia abyssalis 221-F1^T^ (Albuquerque et al., 2015); 5, Palleronia soli CAU 1105^T^ (Kim et al., 2015). +, Positive; w, weakly positive; −, negative; ND, no data available.

The major cellular fatty acids of strain LCG004 (accounting for more than 1.0%) include C18:1 ω7c (55.77%), C19:0 cyclo ω8c (15.78%), C16:0 (13.26%), C18:0 (3.75%), C10:0 3-OH (2.68%), C19:1 ω6c/ω7c/19cy (1.78%), C16:1 ω7c/ω6c (1.4%), C18:1 ω7c 11-methyl (1.29%), C12:0 (1.1%, Supplementary Table 3) The information of fatty acids from other related species is also marked in the table (Martínez-Checa et al., 2005; Albuquerque et al., 2015; Kim et al., 2015; Sun et al., 2021). The polar lipids of strain LCG004 consist of 1,2-dipalmitoyl-sn-glycerol (DPG) and phosphatidylglycerol (PG, Supplementary Figure 1). The respiratory quinone of strain LCG004 was identified as ubiquinone-10 only (Supplementary Figure 2), which is similar with other *Palleronia* species (Martínez-Checa et al., 2005; Albuquerque et al., 2015; Kim et al., 2015; Sun et al., 2021; Barnier et al., 2022).

### 3.2 The genomic features of strain LCG004

**FIGURE 2:**
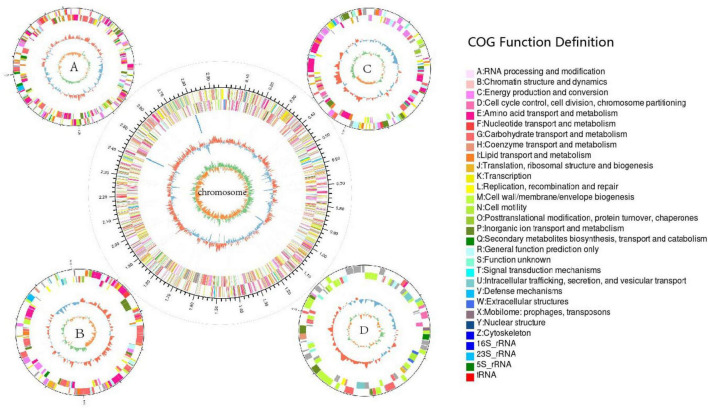
Circular genome map of strain *Palleronia* sp. LCG004. Scale indicates positions in Mbp, starting from the initial coding region. The outermost circle of the ring map is the identification of genome size; the second and third circles are the CDS on the positive and negative strands, with different colors indicating the functional classification of the COGs with different CDSs; the fourth circle is for rRNAs and tRNAs; the fifth circle is for the GC content; and the innermost circle is for the GC-Skew value.

The complete genome of strain LCG004 has a total length of 4,440,293 base pairs, consisting of one chromosome and four plasmids, with a G+C content of 65.06 mol% (Table 2 and Figure 2). The genome encodes 2 homologous ribosomal RNA operons, 48 tRNAs, 48 pseudogenes, and 3,623 predicted protein-coding genes. Among these protein-coding genes, 2,742 genes (75.68%) were assigned to 23 different clusters of orthologous groups (COGs). COG-E (amino acid transport and metabolism), COG-G (carbohydrate transport and metabolism) and COG-R (general function prediction only) are the most abundant COG categories (Supplementary Table 4). Besides, 9 genomic islands and 3 prophage regions were identified in the genome of strain LCG004 (Supplementary Table 5). In addition, 799 proteins (22% of coding genes) within 793 orthologous families were identified in strain LCG004 (Supplementary Figure 3), which have no homolog in other 12 *Palleronia* genomes (BLASTp parameters: identitys, 50; query coverage, 50; and e-value, 1e-5). These pieces of evidences indicated that horizontal gene transfer has played an important role in the evolution of strain LCG004.

**TABLE 2:** Genome features of *Palleronia* sp. LCG004.

Items	Description
Size (bp)	4,440,293
G + C content (%)	65.06
Total Genes	3,728
Protein-coding genes	3,623
Genes assigned to COG	2,742
rRNA operons	2
tRNA genes	48
ncRNA genes	3
Pseudogene	48
Gene islands	9

### 3.3 The phylogeny of strain LCG004

**FIGURE 3:**
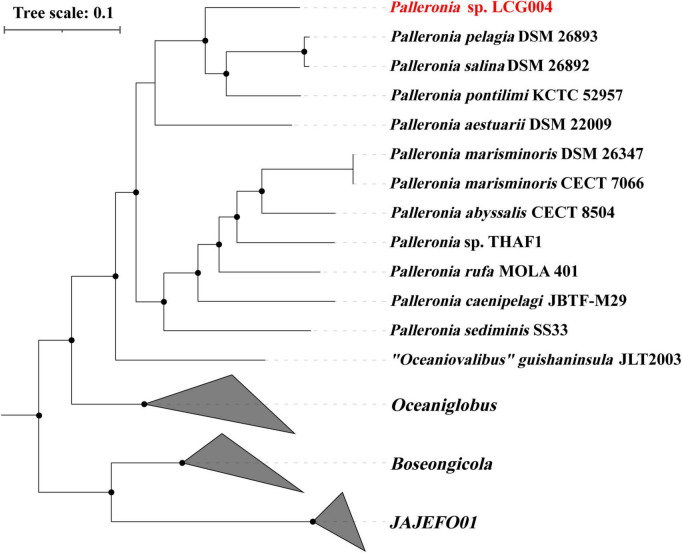
Phylogeny of the *Palleronia* species and their related species based on 120 marked proteins. The *Palleronia* sp. LCG004 is highlighted in red. The origin tree compassed all genera within *Rhodobacteraceae*, and only the branches highly related to *Palleronia* were shown here.

The 16S rRNA gene sequences of strain LCG004 exhibited the highest similarities with those of *Palleronia salina* DSM 26893 (96.96%), *P. salina* DSM 26892 (96.62%) and *P. aestuarii* DSM_22009 (96.46%). Moreover, we constructed a phylogenetic tree based on 120 conserved protein sequences (known as GTDB taxonomy), which encompassed all *Palleronia* genomes from NCBI Refseq database, as well as “*Oceaniovalibus*” *guishaninsula* JLT2003. It showed that strains LCG004 and JLT2003 formed a coherent phylogenetic cluster associated with 11 *Palleronia* strains, demonstrating that they both should be members of genus *Palleronia* (Figure 3). Besides, the average nucleotide identity (ANI) values between strain LCG004 and other strains of *Palleronia* vary from 70.32 to 75.37%, as shown in Supplementary Table 6. These values are below the 95% threshold, which is commonly used to distinguish between bacterial species. Additionally, the digital DNA-DNA hybridization (dDDH) values between LCG004 and *Palleronia* type strains are no more than 18.0% (Supplementary Table 7). These pieces of evidence suggested that strain LCG004 represented a novel species within the genus *Palleronia*.

### 3.4 The metabolic characteristics of strain LCG004

**FIGURE 4:**
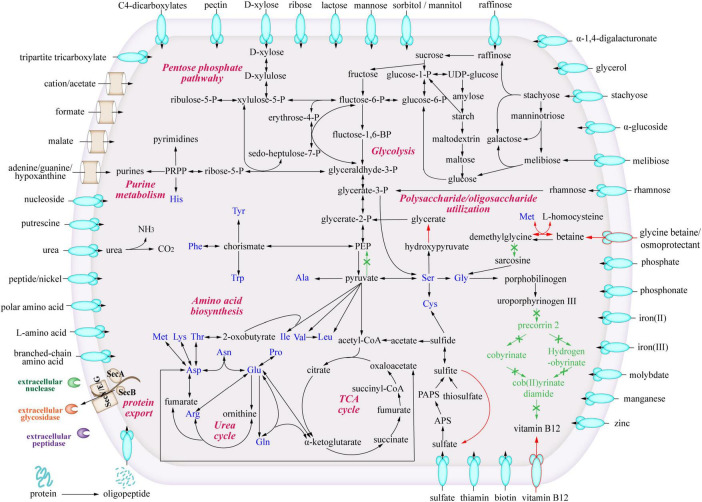
Predicted metabolic pathways of *Palleronia* sp. LCG004. The arrows indicate one or more reactions. Green arrows highlight metabolic pathways missing in strain LCG004 compared to the other 12 closely related *Palleronia* strains, and red arrows show metabolic pathways specific to LCG004.

To study the metabolic characteristics and ecological potentials of strain LCG004, we reconstructed the metabolic pathways and compared them with those of the other *Palleronia* strains (Figure 4).

**FIGURE 5:**
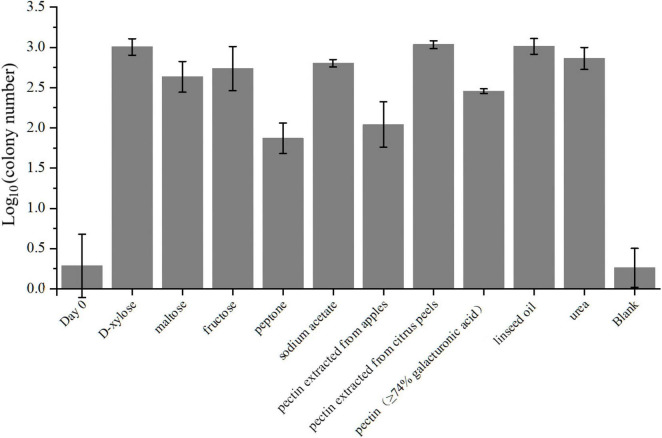
The average colony numbers of strain LCG004 cultivated in different carbon and nitrogen sources. The groups labeled as D-xylose, maltose, fructose, peptone, sodium acetate, linseed oil, pectin isolated from apples, pectin isolated from citrus peels, and pectin with more than 74% galacturonic acid content mean cultivation using either of these compounds as the sole carbon source. The group labeled as “urea” means the cultivation using mixture of D-xylose, maltose and fructose as carbon source, and using urea as the sole nitrogen source. The group labeled as “blank” means the cultivation without adding any carbon or nitrogen source. The bacterial broth after seven-day cultivation was coated on the MB 2216E agar plates for counting the colony number. The group labeled as “day 0” means the average colony number that bacterial broth without cultivation was coated on the MB 2216E agar plates. Three independent biological replicates were performed for each group.

With respect to amino acid metabolism, strain LCG004 possesses a total of 387 COG category E (amino acid transport and metabolism), ranking it second within its genus (only surpassed by *P. aestuarii* DSM 22009 with 409 COG-Es), which indicates that amino acid metabolism is of significant importance to the survival of strain LCG004. In terms of amino acid synthesis, strain LCG004 has the capability to synthesize all 20 essential amino acids. In terms of amino acid metabolism, strain LCG004 possesses a total of 141 genes involved in amino acid and peptide transport (Supplementary Table 8). These include the ABC transport systems of branched-chain amino acids, general L-amino acids, polar amino acids, peptide/nickel, glutathione and oligopeptides, as well as alanine/glycine:cation symporter, sodium/proline symporter and S-adenosylmethionine uptake transporter. Moreover, strain LCG004 contains 9 extracellular (signalP-fused) peptidases (Supplementary Table 9), which play a critical role in the breakdown of peptides into individual amino acids and oligopeptides, thereby facilitating nutrient acquisition. Our experiment showed that strain LCG004 can grow with peptone as the sole carbon source (Figure 5 and Supplementary Figure 4), suggesting that its proteolytic lifestyle could contribute to the organic chemical cycling of amino acids and their derivatives in the intertidal zone.

As for carbohydrate metabolism, strain LCG004 only contains 1 extracellular glycoside hydrolase (RVY76_RS10555, assigned to GH25 family lysozyme). However, it harbors 73 genes involved in transport monosaccharide (e.g., ribose, mannose, gluconate, glycerol, alpha-glucoside and D-xylose), disaccharide (e.g., melibiose, alpha-1,4-digalacturonate, maltose/maltodextrin and lactose), oligosaccharide (raffinose and stachyose), polysaccharides (e.g., pectin, Supplementary Table 8). This suggests that the strain largely relies on the uptake of carbohydrates, followed by their degradation by intracellular glycosidases. Besides the abovementioned carbohydrate utilization tested using API 20 NE, API ZYM strips and GEN III MicroPlates (Supplementary Table 2), our cultivation experiment also confirmed its growth with D-xylose, maltose and fructose as the sole carbon sources (Figure 5 and Supplementary Figure 4).

Specially, pectin is a complex polysaccharide composed of galacturonic acid units and is commonly found in plant cell walls (Khamsucharit et al., 2018). The degradation of pectin involves multiple steps, including redox reactions (Sirotek et al., 2004; Sangeeta et al., 2009). Within the genome of strain LCG004, the genes encoding the pectin ABC transporter subunits (RVY76_RS12725 to RVY76_RS12735) are organized within an operon, along with two Gfo/Idh/MocA family oxidoreductases (RVY76_RS12740 and RVY76_RS12745). These oxidoreductases have the potential to be involved in the oxidation of certain carbohydrate compounds to their corresponding aldonic or carboxylic acids. Such genetic arrangement suggests a coordinated function in the uptake and metabolic processing of complex carbohydrates like pectin. Our experiments further confirmed its growth with either pectin extracted from apples and citrus peels or pectin containing more than 74% galacturonic acid as the sole carbon source, respectively (Figure 5 and Supplementary Figure 4). It highlighted strain LCG004’s potential contribution to nutrient cycling of terrestrial-derived macromolecular organic matter at the interface of marine ecosystems.

In regards to organic acid metabolism, strain LCG004 harbors 53 transporter genes, encoding symporter of cation/acetate, permeases of malate, formate, malonate and short-chain fatty acids, as well as the ABC transport systems of C4-dicarboxylates and tripartite tricarboxylate (Supplementary Table 8). We further confirmed that acetate could support the growth of strain LCG004 as the sole carbon source (Figure 5 and Supplementary Figure 4), indicating that strain LCG004 can also adapt to marine environments where simple organic compounds are available. Moreover, it also processes the genes encoding glycerophosphoryl diester phosphodiesterase and glycerol ABC transport system (Supplementary Tables 8, 9), suggesting its potential to utilize lipids. This hypothesis was further confirmed that it could grow using linseed oil as the sole carbon source (Figure 5 and Supplementary Figure 4). Considering the large amount of lipids released from the lysis of algae after an algal bloom, it suggested that strain LCG004 could aid in the mineralization process of lipid organic matter and play a role in reducing eutrophication in the surface waters of the ocean.

Strain LCG004 possesses a highly versatile capability to utilize nitrogen sources. In addition to utilizing ammonium as an inorganic nitrogen source, it contains as many as six spermidine/putrescine ABC transporter systems (*potABCD* and *potFGHI*, Supplementary Table 8). Specially, we observed one operon, which contains not only *potIHGF* (RVY76_RS00320 to RVY76_RS00335), but also gamma-glutamylputrescine oxidase (RVY76_RS00340, EC:1.4.3.-) and glutamine synthetase (RVY76_RS00345, EC:6.3.1.2). This coordinated regulation likely enhances the efficiency with which strain LCG004 can obtain nitrogen from polyamines. Moreover, it has two urea ABC transport systems (*urtABCDE*) and urease accessory proteins (*ureABCDEFG*). We further confirmed that it could grow with urea as the sole nitrogen source (Figure 5 and Supplementary Figure 4), although the API 20 NE test showed a negative urease activity (Supplementary Table 2). In addition, strain LCG004 harbors the genes encoding a cytosine permease, four general nucleoside ABC transport systems (Supplementary Table 8), as well as three signalP-fused nucleoside/nucleotide hydrolases (Supplementary Table 9), suggesting that the degradation of extracellular nucleic acids is also one strategy by which strain LCG004 can obtain nitrogen. These pieces of evidence indicated that strain LCG004 may have a sophisticated metabolic pathway for the utilization of organic nitrogen compounds, enabling it to efficiently assimilate and metabolize various nitrogen sources for growth and reproduction.

As for phosphor intake, strain LCG004 has four phosphate ABC transport systems, one PiT family phosphate transporter, and one phosphonate ABC transport system (Supplementary Table 8). Besides, it possesses a polyphosphate kinase (RVY76_RS09830), which suggests that the strain could synthesize polyphosphate to restore phosphorus (Supplementary Table 10). This capability could be a strategy for this strain to cope with phosphorus-limited surface seawater environments, thereby ensuring a reserve of this essential nutrient for cellular functions when environmental availability is low.

Strain LCG004 also possesses a highly versatile capability to utilize sulfur sources, including one sulfate/thiosulfate ABC transport system and two sulfate permeases (Supplementary Table 8). Moreover, it exhibits robust metabolic characteristics, including complete pathways for sulfate assimilation and thiosulfate oxidation, evidenced by the presence of the *cysCJHIN* gene cluster and two copies of genes encoding thiosulfate/3-mercaptopyruvate sulfurtransferase (EC:2.8.1.1 2.8.1.2), respectively. Additionally, all other *Palleronia* species employ sulfite dehydrogenase (*soeABC*) to oxidize sulfite to sulfate, transferring electrons to quinone, but this enzyme is strain-specifically absent in strain LCG004. Nevertheless, strains LCG004 can still achieve the oxidation of sulfite, as it contains sulfite oxidase (SUOX, encoded by ILP92_RS17565), which catalyzes the oxidation of sulfite to sulfate using oxygen as the electron acceptor.

With regard to vitamin synthesis, strain LCG004 is capable of producing folate, molybdenum cofactors, and thiamine (although it contains two thiamine ABC transport systems). Like other *Palleronia* species, strain LCG004 cannot synthesize biotin, but relies on a biotin ABC transport system (Supplementary Table 8). It is worth noting that LCG004 strain-specifically lacks almost all genes along the pathway of synthesizing vitamin B12 coenzyme. Alternatively, it possesses a strain-specific vitamin B12 transporter gene (RVY76_RS18495). Actually, the biosynthesis of vitamin B12 requires nearly 30 enzymes (Romine et al., 2017). Therefore, employment of a vitamin B12 transporter rather than biosynthetic pathways may be a strategy for strain LCG004 to save carbon and energy.

### 3.5 Light enhanced the survival and cell aggregation of strain LCG004 under oligotrophic conditions

**FIGURE 6:**
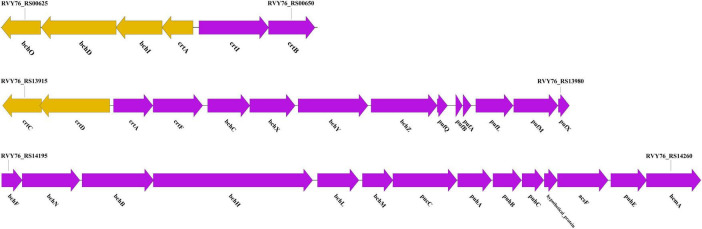
Ge‘ne clusters involved in pigment biosynthesis and photosynthesis within strain LCG004.

We identified four gene clusters involved in pigment biosynthesis and photosynthesis within strain LCG004 (Figure 6 and Supplementary Table 10). The first contains genes for the synthesis of carotenoids (*crtIP* and *hpnCD*). The second encompasses genes crucial for the synthesis of Mg-protoporphyrin IX (*bchIDO*) and the commencement of carotenoid biosynthesis (*crtABI*). The third cluster is replete with genes advancing carotenoid synthesis (*crtCDF*) and bacteriochlorophyll production (*bchCXYZ*), alongside those facilitating the light-harvesting complex assembly (*pufQBALMX*). The fourth cluster harbors genes for the light-independent synthesis of bacteriochlorophyll (*bchFNBHLM*) and essential components of the photosynthetic apparatus, including assembly factors (*pucC, puhABCE*). Notably, only the first gene cluster was identified in *P. marisminoris* CECT 7066, yet the last three clusters are absent therein, corroborating the findings that have not identified bacteriochlorophyll within strain CECT 7066 (Martínez-Checa et al., 2005). Putatively, these genes enable strain LCG004 to synthesize various kinds of pigments to capture and convert a broader spectrum of light energy into chemical energy for growth and metabolism.

**FIGURE 7:**
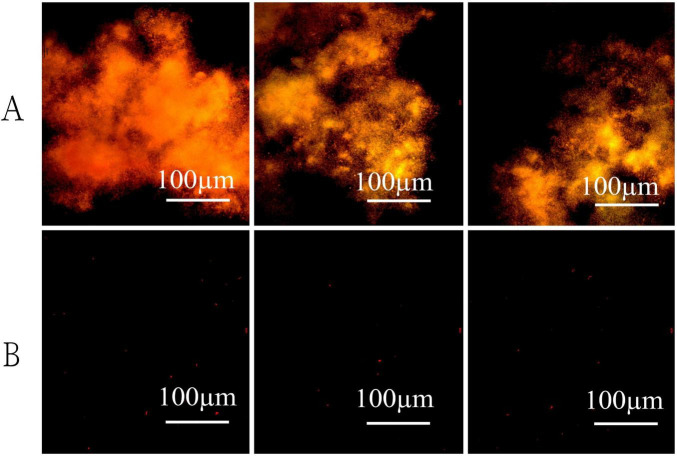
The effect of visible light on bacterial morphology under oligotrophic conditions. Cultures were stained with fluorescent dyes and observed microscopically after 72 h of incubation in light **(A)** and dark **(B)** conditions.

To elucidate the effects of visible light on the growth dynamics of strain LCG004, we conducted experiments under controlled conditions both with and without light. Cultivating the strain for 72 h in nutrient-enriched MB 2216E medium resulted in a pink-hued medium with significant turbidity, yet without any noticeable flocculent particulates. Moreover, there was no significant difference in biomass yield between the light and dark incubation conditions. On the other hand, the oligotrophic medium (containing 1% of the organic components from MB 2216E) incubated in the dark showed a slight turbidity without any visible flocculent particles. In contrast, the oligotrophic medium exposed to light had a significant presence of pale white, suspended flocculent particles. Our subsequent analysis employing the SYTO 9/PI live/dead bacterial double staining method confirmed that the observed particulates were the aggregation of living cells (Figure 7). Conversely, the majority of cells in dark-incubated oligotrophic medium are stained red, indicating a high number of dead cells. These pieces of evidence underscored the significant influence of light on the survival, morphological and physiological attributes of strain LCG004, particularly under oligotrophic conditions. Additionally, we identified two genes (RVY76_RS01660 and RVY76_RS03750) encoding N-acyl-L-homoserine lactone synthetase from the genome, which are signaling molecules used in quorum sensing, a communication system that allows bacteria to coordinate their behavior based on population density. Whether these genes play a role in light-induced cell aggregation still requires further investigation in the future.

### 3.6 The adaptive mechanisms of strain LCG004 to intertidal environments

The intertidal zone acts as a junction among the marine, atmospheric, and terrestrial environments, where the biological communities face fluctuations in factors such as metal ion concentrations, temperature, desiccation, UV radiation, and the force of waves (Wang et al., 2017). Microorganisms in this area experience a dichotomy in their living conditions: they withstand periods of severe, dry, and high-salinity conditions, and alternately flourish in the persistent, salubrious milieu of seawater (Zhang et al., 2013).

Betaine serves as an osmoprotectant that helps microbial cells combat adverse conditions like high salinity, low temperature, high pressure, and desiccation by stabilizing proteins and cell membranes (Carillo et al., 2008; Kim et al., 2009; Mao et al., 2019). Unlike other eight *Palleronia* strains, strain LCG004 together with *P. aestuarii* DSM 22009, *P. pontilimi* KCTC 52957, *P. pelagia* DSM 26893, *P. salina* DSM 26892 was predictively devoid of betaine-aldehyde dehydrogenase (EC:1.2.1.8), which converses betaine aldehyde to betaine. Consequently, they are likely to acquire betaine through active uptake from the surrounding environments. For strain LCG004, in addition to possessing three BCCT family transporters that are involved in the import of compatible solutes such as betaine, choline, glycine, and proline, it also contains a strain-specific ABC transport system (encoded by *opuABCD*) designed for the uptake of osmoprotectants (Supplementary Table 8). The specialized transport system facilitates the efficient and ATP-driven uptake of osmoprotectants, enabling a quick response and adaptation to the dynamic osmotic stress encountered in the intertidal zones’ fluctuating environments.

Betaine degradation also affects the levels of betaine in microbial cells. For instance, glycine betaine monooxygenase (GBMO, EC:1.14.13.251) catalyzes the conversion of betaine into dimethylglycine, which is subsequently transformed into sarcosine by the action of dimethylglycine dehydrogenase (DMGDH, EC:1.5.8.4). We identified GBMO in strain LCG004, as well as in many other *Palleronia* species (Supplementary Table 10). However, DMGDH is strain-specifically absent in strain LCG004, indicating that dimethylglycine cannot be further metabolized. Alternatively, strain LCG004 strain-specifically employs betaine-homocysteine S-methyltransferase (BHMT, EC:2.1.1.5), which mediates a reversible transmethylation reaction that effectuates the metabolic interconversion between betaine and L-homocysteine, and methionine and dimethylglycine (Figure 4 and Supplementary Table 10). We propose that the absence of DMGDH in strain LCG004 may be advantageous for maintaining a relatively stable concentration of betaine within cells. Meanwhile, by regulating GBMO and BHMT expression, strain LCG004 could fine-tune intracellular betaine levels, enabling adaptation to the intertidal zone’s ever-changing conditions.

Furthermore, it was reported that microorganisms may increase the proportion of unsaturated fatty acids in membrane lipids to enhance the fluidity of the cell membrane as a strategy to combat high osmotic and low temperature pressure (Long et al., 2018). As mentioned above, strain LCG004 contains a high proportion of the monounsaturated fatty acid C18:1 ω7c (55.77%, Supplementary Table 3). It suggested that these unsaturated fatty acids may play a crucial role in the intertidal zone survival by maintaining the integrity and fluidity of the cell membrane under fluctuating environmental conditions, such as variations in salinity and desiccation (He et al., 2017).

Moreover, intertidal microorganisms are constantly challenged by the dynamic conditions of their environment, which include not only fluctuations in oxygen levels that can lead to the formation of reactive oxygen species (ROS) such as superoxide anions and hydrogen peroxide but also exposure to sunlight that generates ROS through photochemical reactions (Van Erk et al., 2023). Also, changes in salinity and temperature can disrupt cellular metabolism, resulting in additional ROS production (Xue et al., 2022). To cope with these oxidative stresses, strain LCG004 have evolved with a robust enzymatic defense system, equipped with three catalases, one peroxidase, and three superoxide dismutases (Supplementary Table 10), enabling it to thrive in the intertidal environments.

Additionally, during low tide, microbes exposed to the air are subjected to intense ultraviolet (UV) radiation (Bischof et al., 2000). Strain LCG004 possesses a deoxyribodipyrimidine photo-lyase (RVY76_RS08430), an enzyme capable of repairing pyrimidine dimers through photoreactivation. This capability might allow LCG004 to adapt to the DNA-damaging effects of UV exposure, putatively ensuring its survival and functionality in the challenging intertidal environment.

## 4 Conclusion

In conclusion, the genomic analysis of *Palleronia* sp. LCG004, a bacterium indigenous to the intertidal environment of Lu Chao Harbor, has unveiled a constellation of adaptive mechanisms that underscore its ecological resilience. The strain’s metabolic repertoire encompasses a diverse set of pathways adept at harnessing a spectrum of organic substrates—ranging from simple sugars and organic acids to complex carbohydrates and amino acids—while also effectively managing inorganic nutrient assimilation, pivotal for its role in the ecosystem’s nutrient cycling. Notably, strain LCG004’s capacity for augmenting cell growth and aggregation under oligotrophic conditions, particularly in response to light exposure, highlights the influence of light on its morphological and physiological attributes. The strain’s genomic endowment further includes a suite of osmoprotectant transporters, a deoxyribodipyrimidine photo-lyase for DNA repair, and a sophisticated antioxidant defense system featuring an array of catalases, peroxidases, and superoxide dismutases, collectively conferring resistance to the intertidal zone’s fluctuating conditions, UV radiation, and oxidative stress. This study delineates the multifaceted metabolic capabilities of strain LCG004, illuminating the intricate survival strategies of microorganisms in fluctuating environments and emphasizing their indispensable contribution to global biogeochemical cycles.

## Data Availability

The datasets presented in this study can be found in online repositories. The names of the repository/repositories and accession number(s) can be found below: https://www.ncbi.nlm.nih.gov/, GCF_032931615.1.

## Footnotes

1
https://github.com/dparks1134/comparem
