# Supplementary material for: The metabolic characteristics and environmental adaptations of the intertidal bacterium Palleronia sp. LCG004
Source: Front Microbiol. 2024 Nov 22;15:1469112. doi: 10.3389/fmicb.2024.1469112 (PMC11638410; doi:10.3389/fmicb.2024.1469112)

**Supplementary Tables and Figures:**

**Supplementary Tables:**

**Table S1:** The MIGS morphological information and general characteristics of *Palleronia* sp. LCG004.

**Table S2:** Physiological and biochemical features of strain LCG004 based on API ZYM, API 20 NE, Biolog GEN III.

**Table S3:** Cellular fatty acid contents of strain LCG004 and the related species.

**Table S4:** The COG classification of strain LCG004 and other twelve *Palleronia* strains.

**Table S5:** Genomic island and prophage regions of strain LCG004.

**Table S6:** The ANIb values and aligned percentage between strain LCG004 and other strains.

**Table S7:** The dDDH values between strain LCG004 and other strains.

**Table S8:** Predicted genes related to substrate transport in strain LCG004.

**Table S9:** The extracellular (signalP-fused) peptidases genes predicted in strain LCG004.

**Table S10:** Clusters of genes involved in pigment biosynthesis and photosynthesis and other important genes in strain LCG004.

**Supplementary Figures:**

**Figure S1:** The polar lipids of strain LCG004, including phosphatidylglycerol (PG), 1,2-dipalmitoyl-sn-glycerol (DPG), 4 kinds of unidentified phospholipids (PL1, PL2, PL3 and PL4), 1 kind of unidentified aminolipids (AL), 3 kinds of unidentified glycolipids (GL1-GL3), and 3 kinds of unidentified lipids (L1-L3).

**
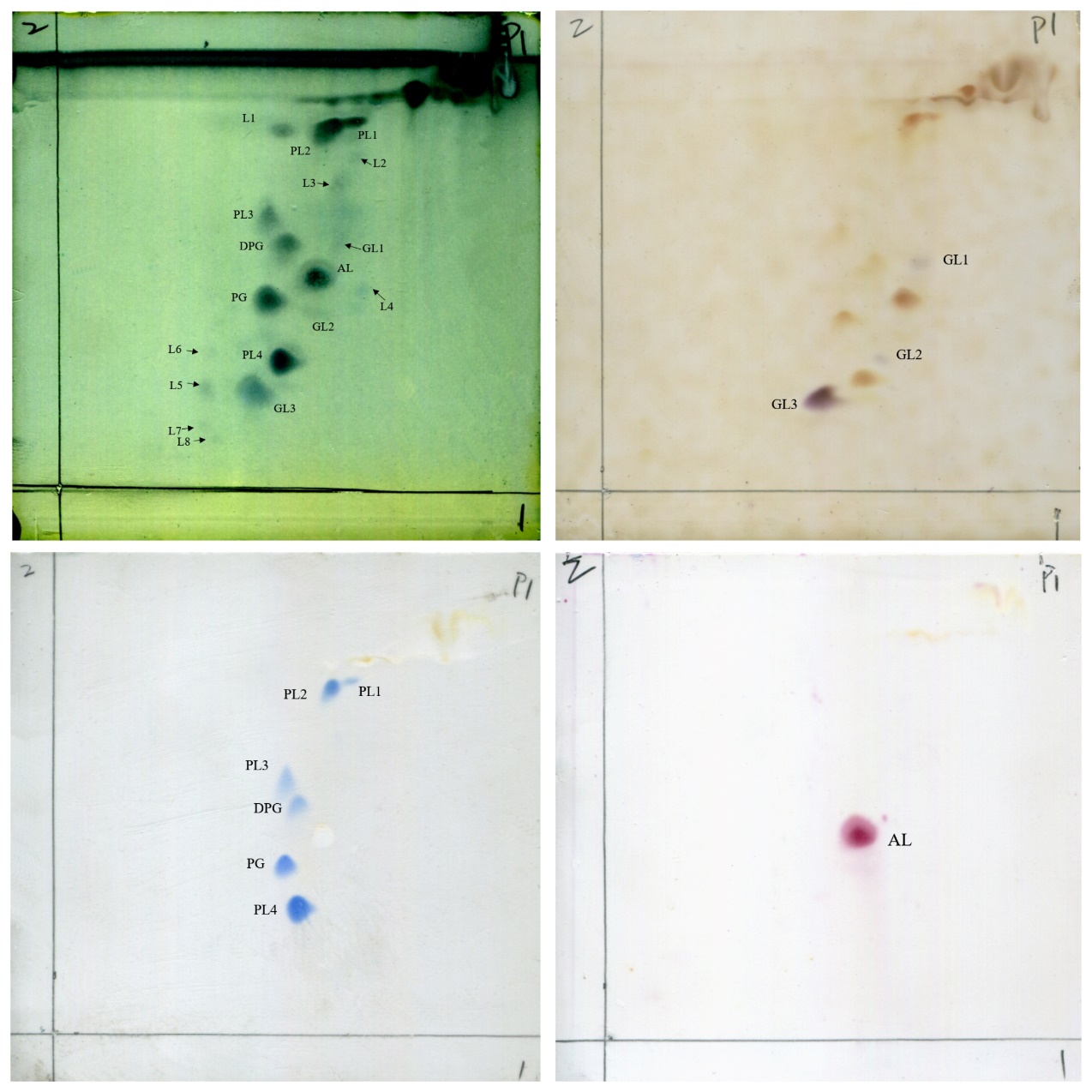
**

# Figure S2: The Total Ion Chromatogram (TIC) and MS Spectra of strain LCG004. The respiratory quinone of the strain was identified as ubiquinone-10 after peak time (RT:24.74) and spectral library comparison.


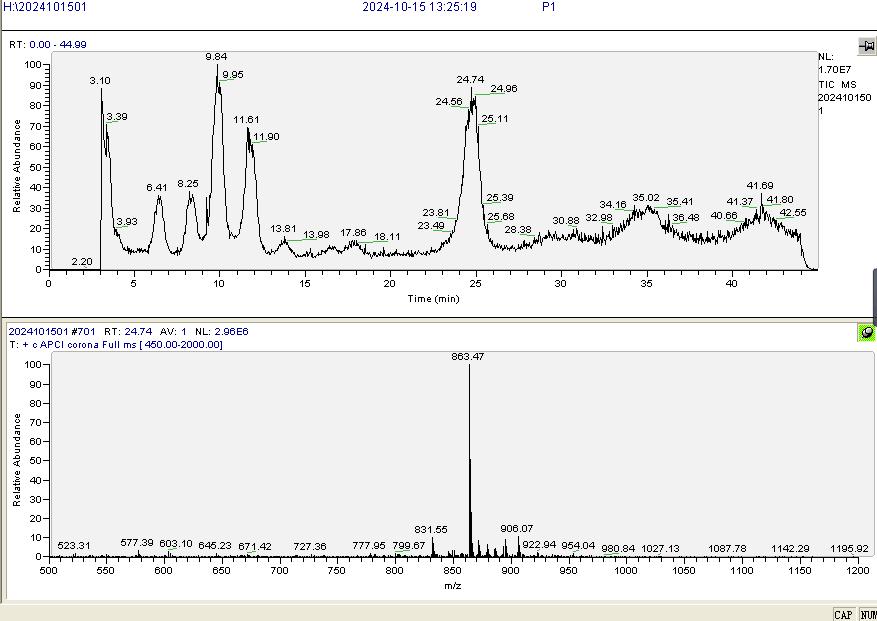


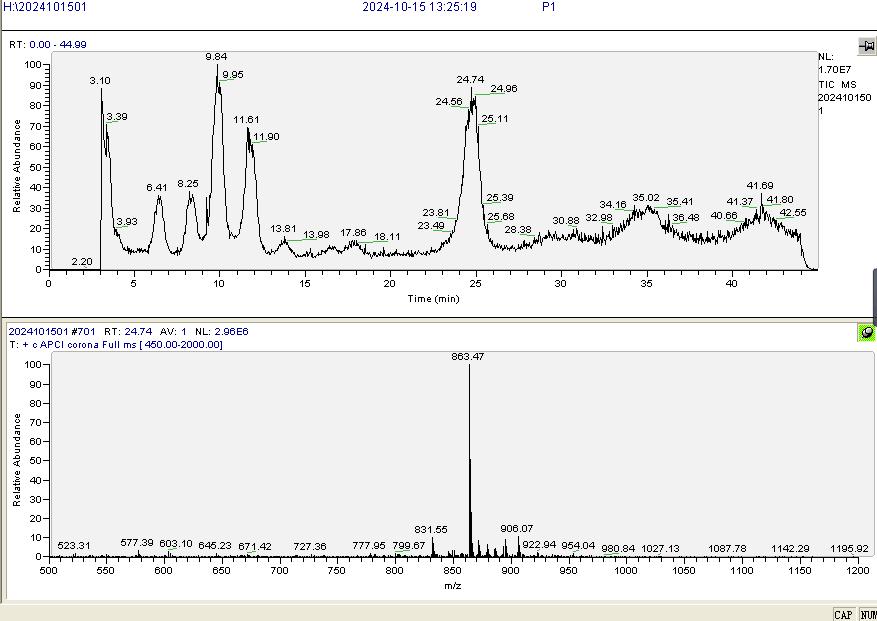


**Figure S3:** Flower plot of exclusive and shared genes for LCG004 and 12 other *Palleronia*.sp strains (BLASTp parameters: identitys, 50; query coverage, 50; e-value, 1e-5).

**
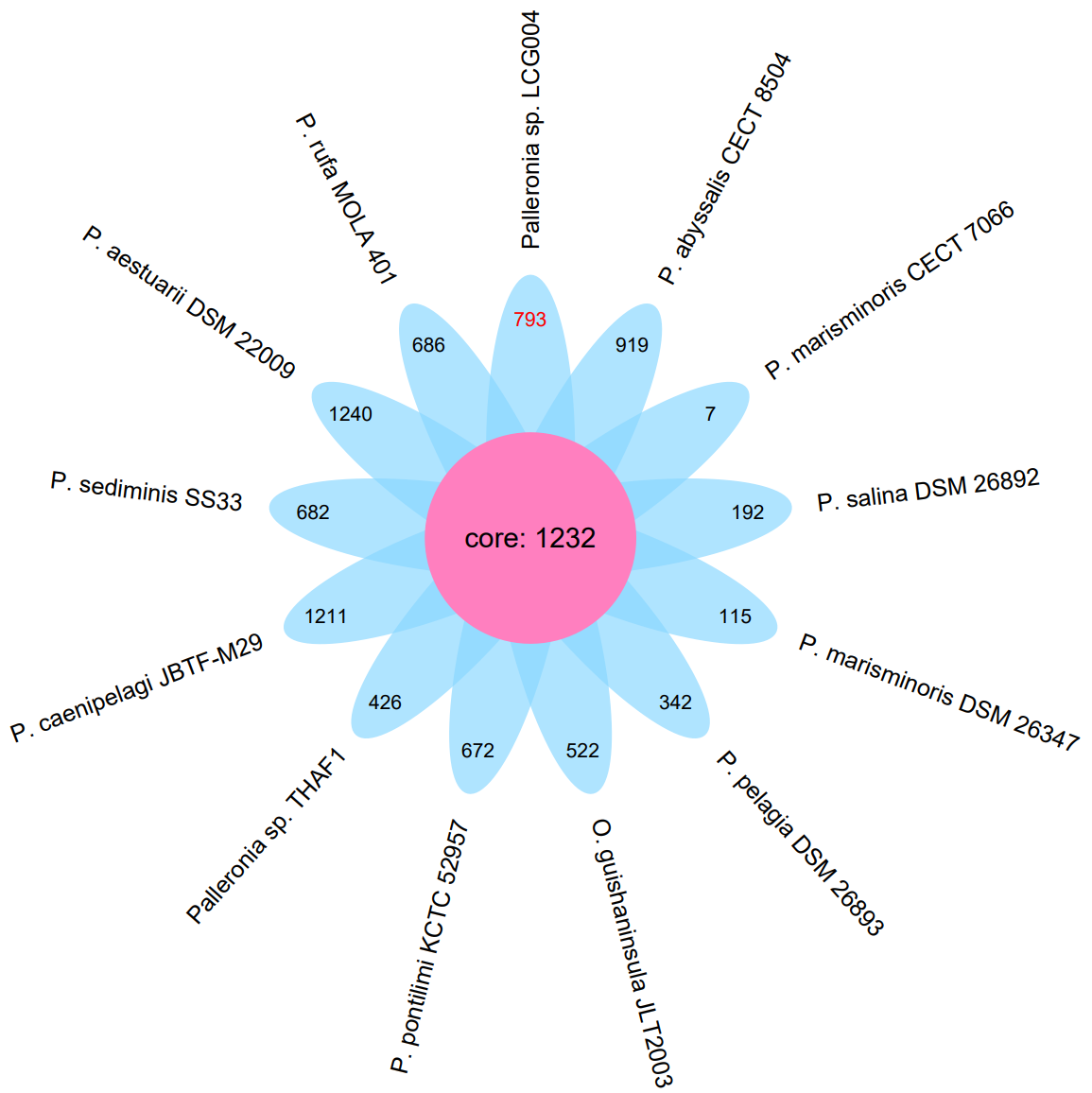
**

**Figure S4:** The colonies of strain LCG004 cultivated with different carbon and nitrogen sources. Strain LCG004 was cultivated in culture medium with (A) D-xylose, (B) maltose, (C) fructose, (D) peptone, (E) sodium acetate, (F) pectin isolated from apples, (G) pectin isolated from citrus peel, (H) pectin with more than 74% galacturonic acid content and (I) linseed oil as the sole carbon source. (J) means the cultivation using mixture of D-xylose, maltose and fructose as carbon source, as well as urea as the sole nitrogen source. (K) represents the blank control without carbon and nitrogen source. The bacterial broth was then coated on the 2216E agar plates at day 0 (before cultivation) and day 7 (after cultivation) for counting colony numbers. Three independent biological replicates were performed for each sample.


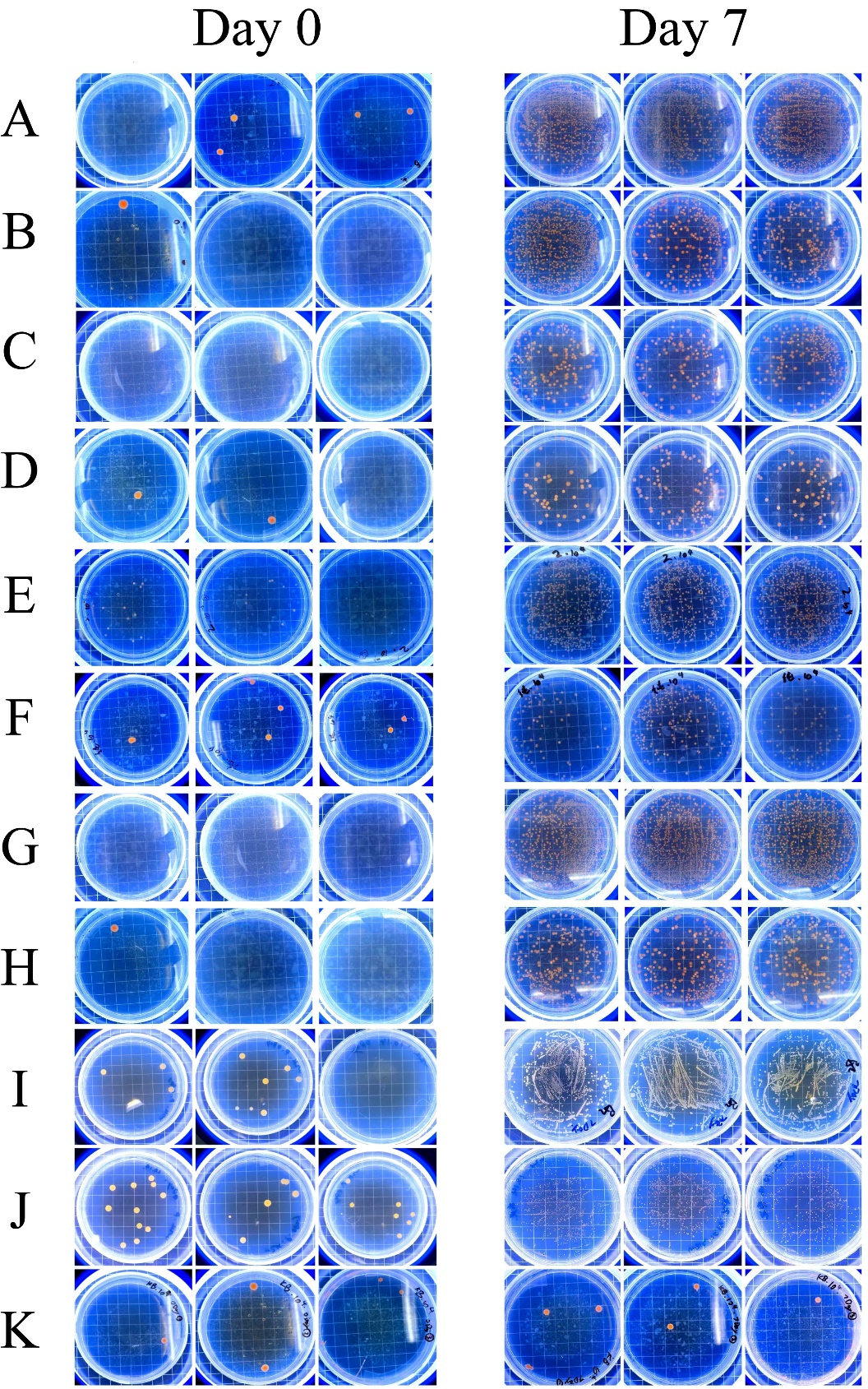

Supplement: Supplementary file 1 [file Supplementary_file_1.docx]
